# A quest for therapeutic antigens in bone and soft tissue sarcoma

**DOI:** 10.1186/1479-5876-3-31

**Published:** 2005-08-08

**Authors:** Satoshi Kawaguchi, Takuro Wada, Tomohide Tsukahara, Kazunori Ida, Toshihiko Torigoe, Noriyuki Sato, Toshihiko Yamashita

**Affiliations:** 1 Department of Orthopaedic Surgery, Sapporo Medical University School of Medicine, Sapporo, Japan; 2 Department of Pathology, Sapporo Medical University School of Medicine, Sapporo, Japan

## Abstract

Over the past three decades, there have been remarkable advances in the treatment of bone and soft tissue sarcomas. These include the introduction of adjuvant chemotherapy, establishment of guidelines for adequate surgical margins, and the development of post-excision reconstruction. There have also been advances in the field of immunotherapy against bone and soft tissue sarcomas, which, unfortunately, have received less attention. However, lack of progress in chemotherapy-based treatments for bone and soft tissue sarcomas has reignited interest in immunotherapeutic approaches. Here we summarize current progress in the immunotherapy of bone and soft tissue sarcomas including the strategies utilized to identify tumor-associated antigens, and the design of clinical trials.

## Introduction

Bone and soft tissue sarcomas are aggressive malignant tumors of mesenchymal origin. Despite their low prevalence (approximately 1% of all malignant tumors), they include more than 60 histological subtypes [1,2]. These subtypes are often associated with unique clinical, prognostic and therapeutic characteristics, leading to difficulties in identifying tumor-specific targets for immunotherapy and/or other therapeutic modalities. In contrast, about 10% of newly diagnosed cancers in children and adolescents constitute rhabdomyosarcoma, osteosarcoma, and Ewing sarcoma, highlighting the weight that these three sarcomas play in pediatric oncology [3].

Until 1970, radical excision was the mainstay of treatment for bone and soft tissue sarcomas. This treatment had resulted in five-year survival rates of 10–20% in patients with osteosarcoma and Ewing sarcoma [3]. To improve such dismal treatment outcomes, efforts have been to foster two distinct approaches; chemotherapy and immunotherapy [4]. Early immunotherapeutic efforts consisted of tumor extracts or whole tumor cells used for direct autologous vaccination [5,6] or allogeneic adoptive immunotherapy [7,8] in patients with osteosarcoma. These limited trials suggested an increase in survival rate over surgery alone. However, the results were not as promising as those of chemotherapy trials conducted during the same period [9,10]. Consequently chemotherapy has to date overshadowed immunotherapy and neoadjuvant chemotherapy in combination with surgery has become the standard treatment of osteosarcoma and other pediatric sarcomas [1,3].

With the technological advancements that lead to the identification of tumor antigens [11], immunotherapeutic approaches shifted from the usage of crude tumor extracts or tumor cells to well-characterized antigenic epitopes. In turn, peptide-based immunotherapy has allowed a more precise monitoring of immunological responses to vaccines. While progress has been made primarily in the context of other tumor models such as melanoma, the relative effectiveness of chemotherapy and practical difficulties in establishing autologous tumor-CTL pairs have delayed the interest in tumor antigen identification out of bone and soft tissue sarcomas [12]. This attitude has perpetuated until recently, when tumor-specific fusion genes resulting from chromosomal translocation were defined in soft tissue sarcomas and later proposed as candidate antigens [13-15]. In addition, the authors' group recently identified an osteosarcoma antigen upon establishment of an autologous tumor-CTL pair [16,17]. These insights have revived the interest to immunotherapeutic approaches to bone and soft tissue sarcomas based on molecularly defined targets.

Current multimodality treatment protocols including surgery, systemic chemotherapy, and radiotherapy yield five-year survival rates around 50–70% in patients with high-grade primary bone and soft tissue sarcomas [1,3]. However, in the majority of cases, disease presenting with metastatic spread or relapses after primary treatment remains incurable, emphasizing the need for alternative treatments of bone and soft tissue sarcomas.

### Immunogenicity of bone and soft tissue sarcoma

#### Clinical observations

Efficacy of immunotherapy depends primarily on the inherent immunogenicity of the corresponding tumors. This can be partly predicted by clinical observations related to the natural behavior of immune responses of the host, such as infilitration of T lymphocytes in tumor tissues and spontaneous tumor regression. Infiltration of lymphocytes has been seen in a group of inflammatory soft tissue sarcomas, including inflammatory myofibroblastic tumors [18] and inflammatory malignant fibrous histiocytomas [19]. However, lymphocytes infiltrating these tumors are considered to be attracted non-specifically by cyotokines produced by tumor cells and not by tumor antigens [20]. Besides inflammatory tumors, tumor-infiltrating lymphocytes (TIL) were sporadically observed in soft tissue sarcomas [21-23]. Notably, infiltration of dendritic cells was also reported in some soft tissue sarcomas with reactive lymphoid hyperplasia [23]. This suggests that presentation of tumor antigens may occur in the regional lymph nodes. To date, prognostic significance of lymphocyte- or dendritic cell-infiltration in bone and soft tissue sarcomas remains to be proved. Also, it remains unknown whether there are differences in the immunogenicity of sarcomas between children and adults.

Spontaneous tumor regression is far more rarely observed. To our knowledge, there are only two reported cases of spontaneously regressing osteosarcoma [24,25]. These observations suggest the relatively low immunogenicity of bone and soft tissue sarcomas in natural conditions. Compared to malignant melanomas that are co-localized with Langerhans cells in the dermis and epidermis, there are few, if any, such immuno-surveillant cells in the deep tissues where sarcomas are developed. Such micro-environmental features may also be attributed to low immunogenicity of bone and soft tissue sarcomas.

#### Laboratory observation

The identification of TIL suggests the question of whether antigen-specific CTL precursors exist in the peripheral blood of patients with bone and soft tissue sarcomas. CTL precursors could home to the tumor site or originate from the presentation of tumor antigens by antigen presenting cells in tumor draining lymph nodes. Two pioneering experimental observations [26,27] answered this question. Ichino et al. [26] induced CTLs from peripheral blood mononuclear cells of six patients with osteosarcoma, which specifically lysed fresh autologous tumor cells. Subsequently, Slovin et al. [27] developed CTL clones from one patient with osteosarcoma, and two patients with malignant fibrous histiocytoma (MFH). We have also recently established three autologous tumor cell-CTL pairs from patients with osteosarcoma [16], MFH of the bone [28], and MFH of the soft tissue, respectively. In our experience, the main difficulty in identifying sarcoma-specific antigens comes from the poor adaptability of sarcoma cells to *in vitro *culture, while the induction of tumor-specific CTLs from peripheral blood mononuclear cells is relatively more reliable thanks for the advancement of techniques for in vitro T cell culture. In addition, the utilization of tumor-draining lymph node as a source of antigen-specific lymphocytes and the manipulation of co-stimulatory pathways such as B7/CD28 and 4-1BB/4-1BBL have enhanced the success rate of CTL induction [28,29]. The efficacy in manipulation of costimulatory pathways to elicit immunological responses *in vivo *has been applied to animal models with osteosarcoma [30-34] and used in clinical trials on patients with malignant melanoma [35,36].

Another important advancement in the characterization of natural immune responses to sarcomas has come from the direct enumeration of tumor antigen-specific T cells in the circulation of patients with cancer. The introduction of HLA/peptide tetramer complexes technology [37] has made it possible to more precisely monitor the presence and frequency of circulating CTL precursors when the antigenic epitope and its associated HLA alleles is known. In this technology, soluble HLA/peptide complexes are assembled and biotinylated in vitro, and then are forced to form tetrameric arrays by addition of fluorescent-labeled avidin molecules. The HLA/peptide tetramer complexes bind to a T cell receptor on lymphocytes, which are visualized by FACS analysis. Using HLA-A24/SYT-SSX peptide tetramers, we observed an increased frequency of CTL precursors specific for SYT-SSX-derived antigenic peptides in patients with synovial sarcomas, compared to those with other sarcomas and healthy donors [38-40]. In addition, increased *in vivo *frequency of CTL precursors recognizing SYT-SSX-derived peptides was significantly associated with present or past history of distant metastases [38]. Thus, aberrantly expressed SYT-SSX gene products are likely to have primed SYT-SSX-specific CTL precursors during the process of systemic tumor spreading. Furthermore, CTLs specific for HLA-A24-positive synovial sarcoma cells were inducible from PBLs of patients who showed increased frequency of CTL precursors in the HLA/peptide tetramer. Together, these findings suggest that SYT-SSX fusion gene products serve as the tumor associated antigen of synovial sarcoma.

### Antigenic peptides in bone and soft tissue sarcomas

#### Derivatives of fusion gene product

Identification of tumor associated antigens and related antigenic peptides is the prerequisite for the development of the antigen-specific peptide-based immunotherapy. The discovery of tumor-specific chromosomal translocation and resultant fusion genes as a common event in various soft tissue sarcomas was a significant aid in antigen identification [13-15]. Since the fusion regions of translocation products are strictly limited to the corresponding tumors [41] (Table 1), they serve as attractive targets for tumor antigen-specific therapies. This concept has been proven previously by the study of leukemias [42,43].

**Table 1:** Chromosomal translocation and fusion gene in bone and soft tissue sarcoma

Tumor	Choromosomal translocation	Fusion gene	Frequency
Synovial sarcoma	t(X;18)(p11.2;q11.2)	SYT-SSX1	>60%
		SYT-SSX2	35%
		SYT-SSX4	rare
Ewing sarcoma	t(11;22)(q24;q12)	EWS-FLI1	85%
	t(21;22)(q22;q12)	EWS-ERG	5–10%
	t(7;22)(q22;q12)	EWS-ETV1	rare
	t(17;22)(q12;q12)	EWS-E1AF	rare
	t(2;22)(q33;q12)	EWS-FEV	rare
Alveolar rhabdomyosarcoma	t(2;13)(q35;q14)	PAX3-FKHR	>75%
	t(1;13)(q36;q14)	PAX7-FKHR	10–15%
Myxoid liposarcoma	t(12;16)(q13:q11)	TLS-CHOP	>75%
Clear cell sarcoma	t(12;22)(q13;q12)	EWS-ATF1	>80%
Dermatofibrosarcoma protuberans	t(17;22)(q22;q13)	COL1A1-PDGFb	>99%

**Figure 1:**
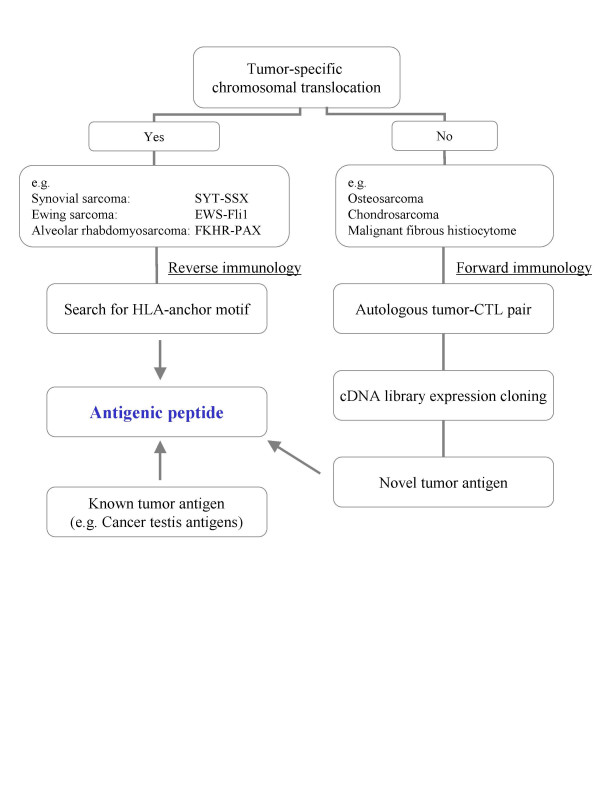
Strategies for the identification of antigenic peptides from bone and soft tissue sarcomas.

A strategic scheme for identification of therapeutic antigens targeting fusion genes is shown in Fig. 1. Soft tissue sarcomas with defined chromosomal translocation may be subjected to search for putative antigenic sequences in the region spanning the fusion gene. This search is based on the recognition of anchor motifs for HLA class I allele commonly expressed by the population affected by the disease [44,45]. This approach is commonly referred to as a "reverse immunology" [46,47]. In this fashion, several antigenic peptides have been defined from SYT-SSX [38,40,48], EWS-FLI1 [49], and PAX3-FKHR [49] fusion gene products (Table 2).

**Table 2:** Antigenic peptide in bone and soft tissue sarcoma

Tumor	Antigen	HLA allele	Peptide sequence*	Reference
Synovial sarcoma	SYT-SSX (SSX1,2,4)	A24	PYGYDQ/IMPK	38
		A24	GYDQ/IMPKK	38,61
		A24	GYDQ/IMPKI	40
		B7	QRPYGYDQ/IM	48
Ewing sarcoma	EWS-FLI1 (type 1)	A3, A31, B44	SSSYGQQN/PSYDSVRRGA	49
	EWS-FLI1 (type 2)	A2, A3, A24, B7, B44, B62	SSSYGQ/QSSLLAYNT	49
Alveolar rhabdomyosarcoma	PAX3-FKHR	A33, A68, B7	TIGNGLSPQ/NSIRHNLSL	49
Osteosarcoma	Papillomavirus binding factor	B55	CTACRWKKACQR	17

Peptide sequences shown to induce cytotoxic T lymphocites or those used in clinical trials are listed. Slash indicates the breakpoint.

#### Derivatives of lymphocyte-defined antigen

Bone and soft tissue sarcomas without known chromosomal translocation may be subjected to the "forward immunology approach" [46] (Fig. 1). This approach is based on the establishment of an autologous pair of tumor cells and tumor cell-reactive CTLs. When the first CTLs were expanded from patients with osteosarcoma [26,27] and MFH [27], the expression cloning procedure for antigen identification [11] had not yet been identified. By using an autologous pair of osteosarcoma cell line and CTL clone [16], we identified an antigenic peptide derived from Papillomavirus binding factor (PBF) [17] (Table 2). PBF was originally defined as a transcriptional regulator of genomic DNA of the human papillomavirus type 8 [50]. The role of PBF in bone and soft tissue sarcomas still remains uncertain. Nevertheless, PBF was found to be expressed in 57/76 cases of bone and soft tissue sarcomas (11/14 osteosarcomas) and 20/34 of epithelial carcinomas by reverse transcription polymerase chain reaction. Immunostaining with anti-PBF antibody was positive in 45/51 bone and soft tissue sarcoma tissues (16/20 osteosarcoma), whereas it was weak and restricted in the cytoplasm of the normal organs tested including ovary, pancreas, and liver [17]. Although PBF could represent a universal target for active specific immunization because of the frequency and relative selectivity of its expression, its utilization is limited by the rarity of the HLA allele in association with whom its discovery occurred. The recognition by the original CTL of the paired autologous tumor cells was associated to HLA-B55. Given the low prevalence of the HLA-B55 allele, the identified antigenic epitope is not clinically applicable to a broad range of patients. Currently the antigenicity of other PBF-derived peptides with high affinity for the HLA-A2 and HLA-A24 alleles, common in both the Japanese and Caucasian population, are being evaluated for future clinical trials.

#### Derivatives of known tumor-associated antigen

In addition to the above described approaches aimed at the identification of novel sarcoma antigens and related epitopes, it is also reasonable to target known tumor-associated antigens that had been previously identified in cancers other than sarcomas as long as these proteins are also expressed by these tumors (Fig. 1). Accordingly, the expression of several cancer-testis antigens [51-54] and other antigenic proteins over-expressed by cancers compared to normal tissues [55,56] has been investigated in bone and soft tissue sarcomas. Despite reasonable expression levels, they have not been proved to be naturally antigenic in patients with bone and soft tissue sarcoma and lead to spontaneous CTL responses identifiable in the blood of these patients. Therefore, peptides derived from those known antigens are now being examined using reverse immunology to test their antigenicity and binding properties to the common HLA alleles, with the aim of utilizing them as targets for antigen-specific immunization.

#### Derivatives of SEREX-defined antigen

SEREX (serological analysis of recombinant cDNA expression library) was developed as a procedure to identify antigens without using autologous tumor-CTL pair [57]. SEREX defines putative antigens using serum IgG antibodies from cancer patients. Since isotype switching from IgM to IgG implies the presence of specific help from CD4+ T cells, antigens defined by SEREX are likely to contain epitopes for CD4+ T cells. SEREX analysis has been performed on patients with bone and soft tissue sarcomas [16,58,59], displaying an array of cancer-testis antigens as possible candidates. However, antigenic peptides have yet to be isolated.

### Clinical trials

To date, various immunotherapeutic trials have been conducted in patients with bone and soft tissue sarcoma (Table 3). They include vaccination of autologous tumors cells [5,6,60], autologous tumor lysates [5,6], antigenic peptide [49,61], and dendritic cells pulsed with tumor lysate [62] or peptides [63]. Also, adoptive transfer of allogeneic lymphocytes sensitized *in vivo *with the recipient's tumor lysate, was reported [7,8]. None of them showed significant adverse events resulting from vaccinations.

**Table 3:** Immunotherapeutic trials in patients with bone and soft tissue sarcoma

Tumor	Clinical setting	Immune intervention	Immunological response	Clinical outcome	Reference
Osteosarcoma	Post-definitive surgery	Autologous tumor cells	N.D	NED at 2y, 1 of 13	5,6
	Post-definitive surgery	Autologous tumor lysate	N.D	NED at 2y, 7 of 16	5,6
	Post-definitive surgery	Allogeneic tumor-sensitized lymphocytes	N.D	NED at 2y, 12 of 32	7,8
Synovial sarcoma	Advanced stage	SYT-SSX peptides-pulsed DC	Cyototoxicity, 1 of 1	PD, 1 of 1	63
	Advanced stage	SYT-SSX peptide	Cytotoxicity, 4 of 5	NC, 1 of 6	61
Ewing sarcoma	Advanced stage	EWS-FLI1 peptide + IL-2	T cell proliferation, 1 of 3	PR, 1 of 12	49
Alveolar rhabdomyosarcoma	Advanced stage	PAX3-FKHR peptide + IL-2	N.D	Response, none of 4	49
Various sarcomas	Advanced stage	Autologous tumor lysate-pulsed DC	DTH, 3 of 10	PR, 1 of 10	62
	Advanced stage	Autologous tumor cell line	DTH, 8 of 16	Response, none of 12	60

DC, dendritic cells; IL-2, interleukin-2; N.D, not determined; DTH, delayed-type hypersensitiveity; NED, no evidence of disease; PD, progressive disease; NC, no change; PR, partial response

Immunological monitoring was not performed precisely in these initial trials due to the lack of target specificity in some trials and the limited expertise in immunological monitoring at that time in which the trails were designed. In contrast, recent trials using fusion gene-derived peptides [49,61,63] associated with specific HLA alleles revealed successful induction of tumor-specific CTLs from five out of ten patients examined, emphasizing the immunological feasibility of this approach. Notably, CTLs specific for a fusion gene-derived peptide could also be identified in only one out of five patients before vaccination in one of our trials [61]. Using HLA/peptide tetrameric complexes, we also noted in this trial, that the frequency of circulating CTL precursors specific to the SYT-SSX peptide increased in three patients while, for unclear reasons, decreased in one patient after vaccination [61].

Therapeutic efficacy cannot be assessed in such exploratory studies including a limited number of patients. However, it is interesting to report that during the conduct of recent clinical trials [49,60-63] tumor remission was observed in two out of a total of 45 patients who had received vaccination; one with Ewing's sarcoma [49] and the other with fibrosarcoma [62]. In these trials, all patients had advanced disease in contrast to the initial trials that had been conducted, in adjuvant setting, in patients with osteosarcoma [5-8]. In the latter trials, the patients underwent definitive amputative surgery for the primary tumor and had no detectable metastasis before immunotherapy. The clinical efficacy of immunological interventions was, therefore, evaluated in its ability to prevent recurrence of disease which most commonly occurs in the lungs as distant pulmonary metastases. In these clinical trials chemotherapy was not given in combination with immunotherapy since at that time there was no evidence that chemotherapy is associated with clinical benefit in adjuvant settings. Interestingly, osteosarcoma has biological features which suite the design of adjuvant immunotherapy trials. These include the frequent existence of micrometastases on initial presentation and a tendency to relapse during the first year of treatment, especially after excision of the primary tumor [64,65]. The disease-free survival at two years was 33% in 32 patients who had undergone adoptive immunotherapy [8], and 44% in 16 patients vaccinated with autologous tumor lysate [6]. In a historical control including 145 patients treated by surgery alone, the two-year disease free survival was as low as 22% [8], suggesting that these immunotherapeutic trials hold some promise. Due to their small size and the lack of an appropriate contemporary and matched control population the significance of these results, however, needs to be further assessed. Nevertheless, the observations from these initial trials suggest that in patients with osteosarcoma and other bone and soft tissue sarcomas vaccination in adjuvant settings would be more likely to yield clinical benefit than vaccination with therapeutic purposes.

## Conclusion

Given the rarity, relative low immunogenicity, and their heterogeneity, bone and soft tissue sarcomas have remained for a long time at the boundaries of tumor immunology. Up to three decades ago, they were perceived similarly by oncologists who inclined to approach cancer with chemotherapy [66]. As chemotherapy has relied upon intensification and multiplication of therapeutic schemes and the production of new chemotherapeutics to successfully enhance its efficacy, tumor immunologists will face the challenge of rationally expanding the use of antigens-specific immunization to enhance its usefulness against bone and soft tissue sarcomas.

## Abbreviations

CTL; cytotoxic T lymphocyte, HLA; human leukocyte antigen
